# Impact of *Staphylococcus aureus* colonization and skin abscesses on formation of human anti-αGal antibodies

**DOI:** 10.1007/s00430-025-00833-3

**Published:** 2025-05-13

**Authors:** Jens Magnus Bernth Jensen, Khoa Manh Dinh, Lotte Hindhede, Lise Tornvig Erikstrup, Annette Gudmann Hansen, Kirstine Mejlstrup Hymøller, Sisse Rye Ostrowski, Ole B. V. Pedersen, Stig Hill Christiansen, Uffe B. Skov Sørensen, Steffen Thiel, Christian Erikstrup

**Affiliations:** 1 Department of Clinical Immunology, Aarhus University Hospital, Aarhus, Denmark; 2 Department of Molecular Medicine, Aarhus University Hospital, Aarhus, Denmark; 3 Department of Clinical Immunology, Copenhagen University Hospital, Rigshospitalet, Copenhagen, Denmark; 4 Department of Clinical Microbiology, Aarhus University Hospital, Aarhus, Denmark; 5 Department of Biomedicine, Health, Aarhus University, Aarhus, Denmark; 6 Department of Clinical Medicine, Faculty of Health and Medical Sciences, University of Copenhagen, Copenhagen, Denmark; 7 Department of Clinical Immunology, Zealand University Hospital, Køge, Denmark; 8 Department of Clinical Immunology, Department of Molecular Medicine, Aarhus University Hospital, Palle Juul-Jensens Boulevard 99, Aarhus N, 8200 Denmark

**Keywords:** Naturally occurring antibodies, Anti-αGal antibodies, *Staphylococcus aureus* colonization, and skin abscesses

## Abstract

IgG antibodies against terminal galactose-α-1,3-galactose (anti-αGal antibodies) are naturally occurring in humans, but their origins remain poorly understood. These antibodies target various microorganisms including *Staphylococcus aureus*, a common nasal commensal and the major cause of skin abscesses. This study investigates the impact of *S. aureus* colonization and abscess events on plasma anti-αGal antibody levels. We measured plasma anti-αGal antibody levels using a quantitative immunoassay in: (i) 101 pairs of healthy individuals with and without nasal *S. aureus* colonization, (ii) 106 healthy individuals before and after abscess formation, and (iii) 43 patients with recurrent skin abscesses compared with 75 patient controls and 60 healthy controls. We observed a 35% reduction (95%CI: 7–54%) in anti-αGal antibody levels in nasal *S. aureus* carriers. Conversely, we found a 30% increase (95%CI: 4–66%) in individuals within 187 days post-skin abscess, and patients with recurrent skin abscesses exhibited 81% higher (95%CI: 14–190%) levels than patient controls, and 110% higher (95%CI: 39–230%) than healthy controls. This study suggests that skin abscesses lead to elevated plasma anti-αGal antibody levels and that these antibodies might convey or correlate with mucosal immunity to *S. aureus*.

**Supplementary Information:**

The online version contains supplementary material available at 10.1007/s00430-025-00833-3.

## Introduction

In scientific literature, the term ‘naturally occurring antibodies’ refers to various antibody fractions characterized in different ways. Here, we use the definition: antibodies against non-self antigens that develop naturally during the first years of life in essentially all healthy individuals, regardless of vaccination status or specific known infections. These antibodies constitute a significant portion of human plasma immunoglobulins and likely provide crucial protection against microorganisms [1–3]. The mechanisms leading to the production of these antibodies are not well understood. Clarifying these mechanisms is of fundamental immunological interest and may aid in the development of future antimicrobial therapeutics.

Naturally occurring antibodies include, for example, those directed against ABO blood group antigens, N-glycolylneuraminic acid, and terminal galactose-α-1,3-galactose (αGal) [4, 5]. Anti-αGal antibodies are a pertinent model for studying the origin of naturally occurring antibodies, as these antibodies are well characterized and among the most abundant in human plasma.

Anti-αGal antibodies of the immunoglobulin classes IgA, IgG, and IgM are present in human plasma [6–8], with IgG anti-αGal antibodies averaging about 10 mg/L [9, 10]. The concentration is generally stable over time within an individual but can vary more than 400-fold between individuals [11, 12]. Some of this variation can be attributed to host factors such as sex, age, and the expression of the ABO-blood group B-antigen [13–15]. The B-antigen (galactose-α-1,3[fucose-α-1,2]-galactose-R), expressed by approximately 10% of humans, structurally resembles αGal. B-antigen expression may reduce the ability to form anti-αGal antibodies due to partial tolerance [16]. However, each of these host factors accounts for at most a two-fold difference in antibody concentrations [17]. Additional factors, such as differential bacterial exposure, are likely to influence individuals´ level of anti-αGal antibodies.

Several lines of evidence suggest that bacteria contribute to the development of anti-αGal antibodies. For example, these antibodies bind to a broad range of bacteria, including both commensals and pathogens [1, 18–21]. Moreover, animal studies have demonstrated that plasma anti-αGal antibody levels increase following oral ingestion of bacteria that react with these antibodies [22], and decrease following bacterial eradication with antibiotics [23]. However, direct evidence from human studies to support this hypothesis is lacking.

Our aim was to investigate the impact of bacterial exposure on human IgG anti-αGal antibody levels in plasma. We selected *Staphylococcus aureus* as a model organism for two reasons. First, anti-αGal antibodies bind to most *S. aureus* strains isolated from humans [20]. Second, *S. aureus* is significant both as a commensal and as a pathogen, especially in relation to skin abscess formation. It is found in approximately three-fourths of skin abscesses [24, 25] but is more rarely implicated in abscesses at other anatomical sites, such as the anal region [26–29]. This differential distribution of *S. aureus* in abscesses helps to control potential confounding factors in our study.

In this study, we measured plasma IgG anti-αGal antibodies in: (i) healthy individuals with and without *S. aureus* nasal colonization, (ii) healthy individuals before and after abscesses at different anatomical locations, and (iii) patients with recurrent skin abscesses compared to patients with non-*S. aureus* infections and healthy controls.

## Materials and methods

### Study populations

#### Three cohorts of participants were studied

***S. aureus*Carriage Cohort**: Participants for this cohort were drawn from the Danish Blood Donor *Staphylococcus aureus* Carriage Study (DBDSaCS) initiated in 2014 as a substudy of the Danish Blood Donor Study (DBDS). DBDS is a nationwide prospective public health study initiated in 2010 [30]. Participants’ anterior nares were swabbed, and the nasal samples were cultured and analyzed by matrix-assisted laser desorption/ionization time-of-flight mass spectrometry as previously described [31]. Only participants from the Central Denmark Region who enrolled before September 30, 2021, were eligible (*n* = 6,391). Among these, participants with two swabs taken on separate occasions were identified (*n* = 2,271) and then categorized as follows: non-carriers (those with two negative nasal samples), intermittent carriers (those with a positive result in one of the two samples), or persistent carriers (those with two positive samples). Two positive swabs accurately predict persistent carrier status [32]. We only included persistent carriers (hereafter referred to as carriers) and non-carriers. Ethylenediaminetetraacetic acid (EDTA)-stabilized plasma samples for anti-αGal antibody quantification were collected at the first nasal swab. For each carrier, a matched non-carrier was assigned based on ABO blood group, age, sex, and availability of a plasma sample donated within 30 days of the carrier´s sample. EDTA-stabilized plasma samples for anti-αGal antibody quantification were collected along with one of these swaps.

**Single Abscess Cohort**: This cohort comprised healthy blood donors selected from the DBDS. We included participants from the Central Denmark Region enrolled before September 30, 2021. Cases were defined by the following criteria: (i) at least one abscess diagnosis (details provided below), (ii) provision of at least one plasma sample within two years prior to the first abscess diagnosis (baseline sample), and (iii) provision of at least one plasma sample between two weeks and two years after the first abscess diagnosis (follow-up sample). The sample closest to the abscess diagnosis was chosen if multiple samples were available. Abscess diagnoses were identified for all eligible donors (*n* = 38,088, Supplementary Table 1) from the Danish National Patient Register (NPR), which contains nationwide information on hospital in- and out-patient contacts from 1995 onwards [33]. For each case, a matched control was assigned based on ABO blood group, age, sex, and availability of a plasma sample donated within 30 days of the case´s baseline sample. Controls had no abscess diagnoses recorded in the NPR. Archived EDTA-stabilized plasma samples were collected between January 1, 2009, and September 30, 2021. Microbial diagnostic data was unavailable for this cohort.

**Recurrent Skin Abscess Cohort**: This cohort was recruited from a previous study of patients with suspected primary immunodeficiency [1]. We included a subgroup of 43 patients with recurrent skin abscesses as their primary reason for referral. Microbial diagnostic data was unavailable for this group. Two control groups were included. **Patient controls**: 75 patients from the original cohort with idiopathic infections other than skin abscesses and airway infections. Airway infections, the primary manifestations of antibody incompetence, were associated with low plasma levels of anti-αGal antibodies in the original cohort [1]. Reasons for referral included viral infections (*n* = 35), invasive bacterial infections (excluding *S. aureus*) (*n* = 16), fungal infections (*n* = 10), and miscellaneous other infections (excluding *S. aureus*) (*n* = 14). The exclusion of individuals with *S. aureus* infections was done to minimize potential confounding effects related to the bacterium under investigation. **Healthy controls**: 60 healthy blood donors included from the previous study [1].

Data on antibiotic exposure was not available for this study.

#### Quantification of plasma anti-αGal antibody

Plasma IgG anti-αGal antibody was quantified using a time-resolved immunofluorometric assay (TRIFMA) [34]. Each sample was applied in duplicate to wells coated with Galα(1–3)Gal conjugated to human serum albumin (HSA) (NGP2203, Dextra Laboratories, UK) and to wells coated with HSA. Bound IgG was detected using biotin-labeled rabbit anti-human IgG antibody (Dako, Denmark), followed by europium-labeled streptavidin and measurement of time-resolved fluorescence on a VICTOR X5 multilabel plate reader (PerkinElmer, MA, USA). For each sample, the signal from the Galα(1–3)Gal-HSA surface was corrected by subtracting the signal from the HSA surface to account for background IgG anti-HSA antibodies [17]. Corrected signals were converted to concentration estimates using standard curves generated from a selected standard sample with a known IgG anti-αGal antibody concentration. Results for unknown samples were expressed relative to the standard sample, using its known IgG anti-αGal antibody concentration as a reference. Potential differences in antibody avidity were not studied. Samples from cases and their associated controls were analyzed together, and the operator was blinded to the sample identities.

#### Statistics

**Fig. 1:**
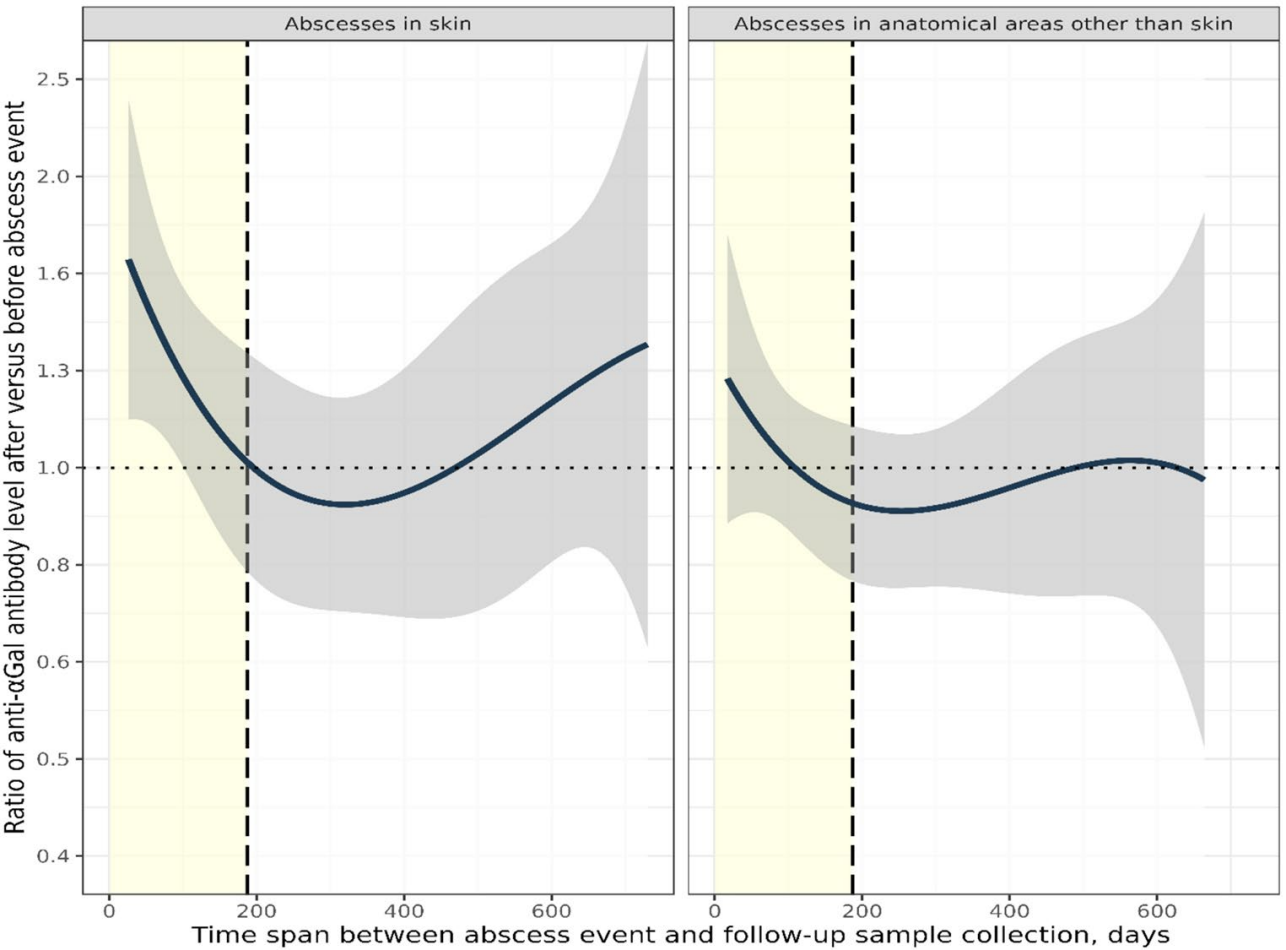
Ratio of plasma anti-αGal antibody concentrations measured after versus before an abscess event, as a function of time from the event to the follow-up measurement. The plot shows the fold-increase in plasma anti-αGal antibody concentration after abscess diagnosis compared to before, plotted against the number of days from abscess diagnosis to follow-up sample collection. A third-order polynomial regression line (black) represents the data, with the grey shaded area indicating the 95% confidence interval. The yellow shaded area represents follow-up time from day 17 to day 187. The vertical long-dashed line indicates the 187-day follow-up time. The horizontal dotted line represents a value of y=1, indicating no difference in antibody level after versus before abscess event. The analysis is stratified by abscess type, with skin abscesses on the left and non-skin abscesses on the right. Please note that the y-axis parameter (ratio of paired antibody concentrations) is unitless. Individual data points are omitted to adhere to data protection regulations

**Fig. 2:**
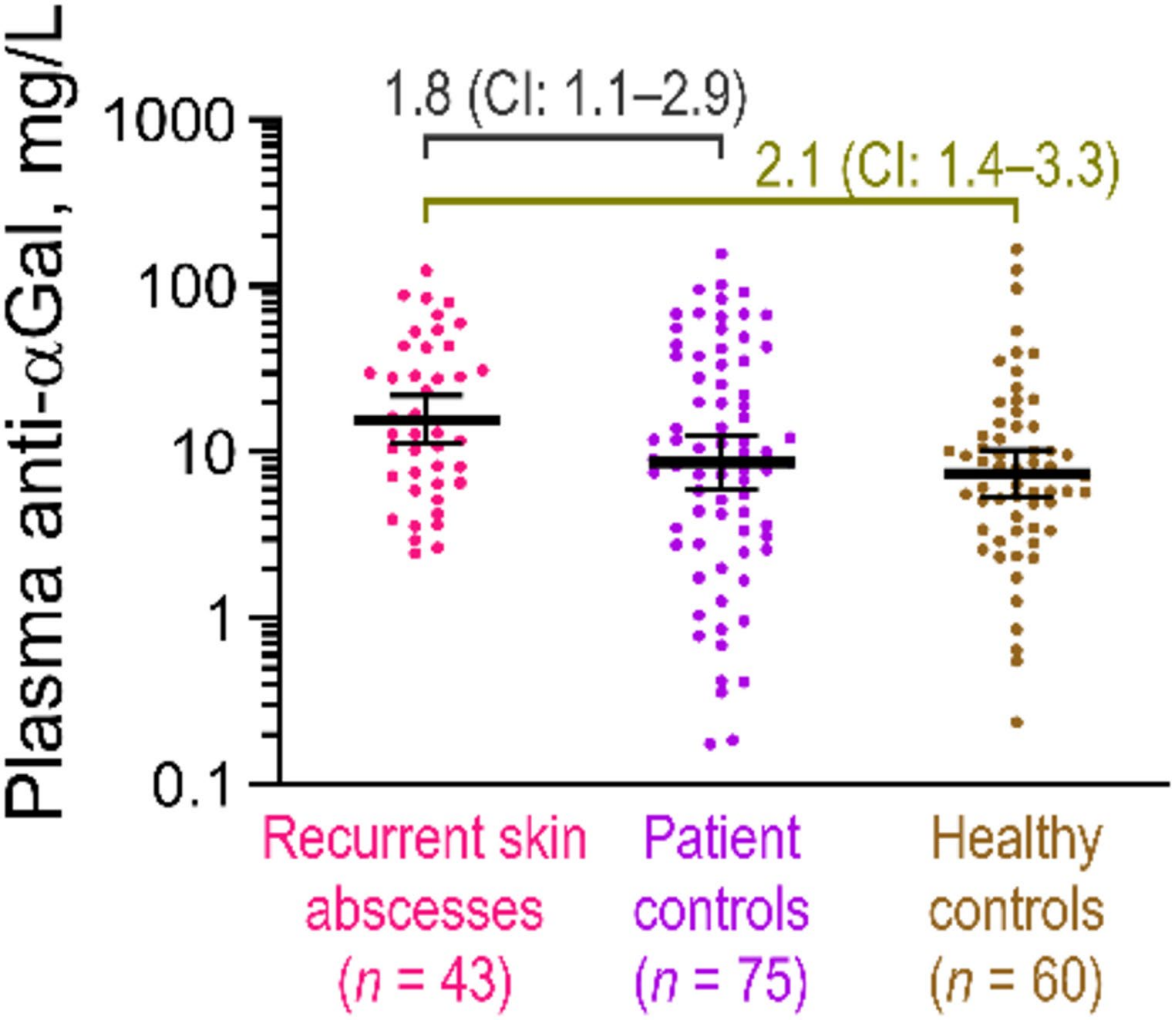
Plasma anti-αGal antibody concentrations in patients with multiple cases of skin abscesses and controls. Anti-αGal antibody concentrations were measured in plasma samples using a solid-phase immunoassay. This substudy included three groups: Patients with a history of recurrent skin abscesses to a degree prompting referral for immunodeficiency testing (n = 43), Patient controls who were referred for immunodeficiency testing due to increased susceptibility to infections, excluding airway infections or infections by *S. aureus* (n = 75), and healthy blood donor controls (n = 60). Anti-αGal antibody levels in patients with recurrent skin abscesses were compared to the two control groups using two-sided permutation tests with 5000 iterations. Black bars represent geometric means with 95% confidence intervals. The relative average antibody concentrations between cases and controls are shown with 95% confidence intervals (CIs)

**Fig. 3:**
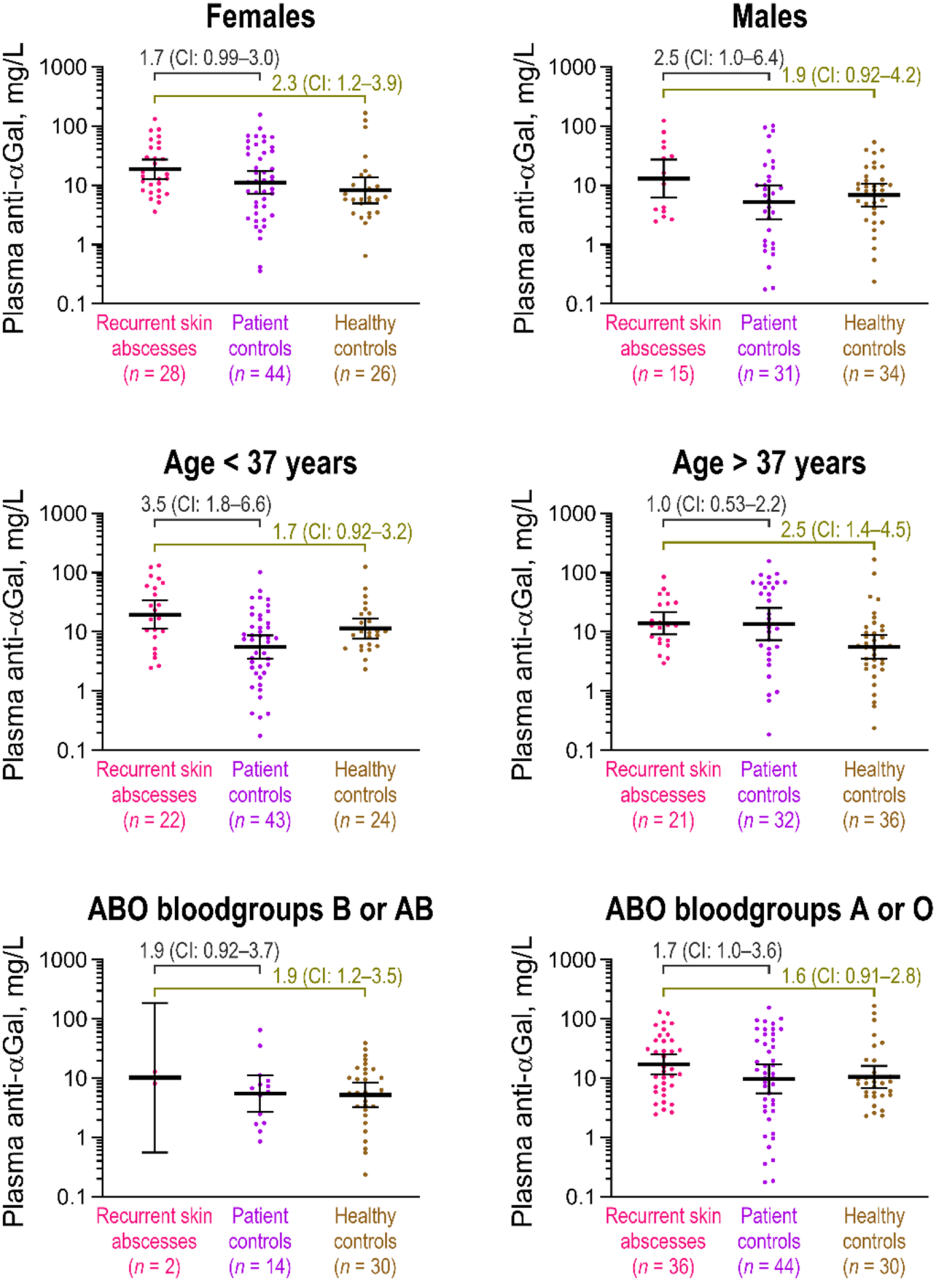
Stratified analyses of plasma anti-αGal antibody concentrations in patients with multiple skin abscesses and controls. Same data as in Fig. 2, now stratified by sex (top row), age below or above the median of all individuals included in the recurrent skin abscess cohort (median: 37 years) (middle row), and B-antigen expression (bottom row). The relative average antibody concentrations between cases and controls are shown with 95% confidence intervals (CIs). ABO blood group data were available for 38 cases and 58 patient controls but were complete for all healthy controls

The data from the Recurrent Skin Abscess Cohort were analyzed as independent samples using the online tool ‘Estimation Statistics’ [35]. Data from the other two cohorts were analyzed as dependent samples in ‘R’ (version 4). For data organization and the construction of Fig. 1, the tidyverse package was employed. Effect sizes with 95% confidence intervals (95% CI) were calculated from the distribution of 5000 bootstrap samples. Statistical significance was determined by two-sided permutation tests with 5000 iterations. Custom R code was used for bootstrapping and permutation analysis. P-values < 0.05 were considered statistically significant. For DBDS participants, results are not shown for groups with fewer than 5 observations due to data protection regulations. Likewise, individual data points could not be displayed to ensure compliance with these regulations. Figures 2 and 3 were prepared in GraphPad Prism version 10 for Windows (GraphPad Software, Boston, MA, USA).

#### Ethics

The Scientific Ethical Committee in Central Denmark Region approved the DBDS (1-10-72-95-13), the DBDSaCS (1-10-72-307-13), and the study on participants included in the Recurrent Skin Abscess Cohort (1-10-72-127-12). Approvals were also obtained from the Danish Data Protection Agency (P-2019-99, 704157, and 1-16-02-40-12/2007-58-0010). DBDS participants provided informed consent. Patient samples were obtained for the diagnosis of immunodeficiencies, and a waiver of informed consent was granted by the local ethics committee in accordance with Danish health legislation.

## Results

### *S. aureus* nasal carriage is associated with lower anti-αGal antibody levels

To assess the effect of *S. aureus* as a commensal, we compared plasma anti-αGal antibody levels between individuals who were carriers or non-carriers of this bacterium in the *S. aureus* Carriage Cohort. Carriers and non-carriers were individually matched for sex, age, and ABO blood group type. Anti-αGal antibodies were quantified in plasma samples from all available matched pairs (*n* = 101) using a quantitative immunoassay.

We found that the average plasma concentration of anti-αGal antibodies was 10 mg/L (95%CI: 8.1–13 mg/L) in carriers and 16 mg/L (95%CI: 12–21 mg/L) in non-carriers (Table 1). Paired analysis revealed that carriers had, on average, 35% (95%CI: 7–54%) lower anti-αGal antibody concentrations compared with non-carriers. Similar results were observed when stratified by sex, age, and B-antigen expression (Table 1).

**Table 1:** Plasma anti-αGal antibody concentration in healthy nasal carriers or non-carriers of *S. aureus*

		Mean anti-αGal in mg/L (95% CI)			
	**Pairs**, ***n***	**Carriers**	**Non-carriers**	**Concentration ratio** **(95% CI)**	**P**
All	101	10 (8.1, 13)	16 (12, 21)	0.65 (0.46, 0.93)	0.02
Sex stratified:					
Females	33	6.1 (4.4, 8.4)	10 (7.6, 14)	0.58 (0.39, 0.91)	0.02
Males	68	13 (9.9, 18)	19 (13, 28)	0.69 (0.42, 1.15)	0.15
Age stratified:					
20–34 years	50	10 (7.5, 14)	16 (12, 22)	0.65 (0.41, 1.02)	0.06
35–64 years	51	10 (7.2, 14)	16 (9.5, 24)	0.65 (0.37, 1.19)	0.16
ABO blood group stratified					
B or AB	< 5	N/A	N/A	N/A	
O or A	> 96	10 (8.2, 13)	16 (12, 21)	0.65 (0.45, 0.94)	0.02

The 95% confidence intervals (95% CI) were calculated by bootstrap with 5000 iterations. The p-values represent the likelihood that the average concentration ratio between carriers and non-carriers differs from 1, and were calculated using a two-sided permutation test with 5000 iterations. N/A: Data unavailable (presentation not allowed as less than 5 individuals are included)

### Anti-αGal antibody concentration increases temporarily after skin abscess

To investigate the impact of *S. aureus* infection on plasma levels of anti-αGal antibodies, we established the Single Abscess Cohort (*n* = 106) from a population of 38,088 eligible healthy blood donors. This cohort included individuals with an abscess diagnosis who had provided plasma samples both before and after the abscess event. Follow-up samples were collected between 17 and 730 days after the abscess diagnosis, with a median follow-up time of 187 days.

We found that the average plasma anti-αGal antibody concentration before the abscess event was 11 mg/L (95% CI: 8.4–15 mg/L), which increased marginally to 12 mg/L (95% CI: 9.3–16 mg/L) after the occurrence of an abscess (Table 2). Paired analysis revealed that the ratio of antibody concentrations after abscess formation to those before averaged 1.1 (95% CI: 0.97–1.2), indicating an estimated 10% increase in antibody levels; however, this difference was not statistically significant as the confidence interval included 1.0. We further analyzed the association with sex, age, B-antigen expression, abscess location, and follow-up time. Follow-up time was stratified at the median of 187 days to ensure balanced group sizes, maximize statistical power, and minimize the influence of extreme values. These stratified analyses revealed a statistically significant increase in antibody levels for cases with follow-up times of less than 187 days. In this subgroup, the antibody ratio was 1.2 (95% CI: 1.0–1.4), reflecting a 20% increase. Stratification by follow-up time consistently showed higher point estimates of effect size for the shorter follow-up period (17 to 186 days) compared with the longer period (187 to 730 days) across all subgroups (**Supplementary Table 2**). The increase was statistically significant only in the subgroup with a follow-up time under 187 days and skin abscesses, where the ratio was 1.3 (95% CI: 1.0–1.7), indicating a 30% increase. The point estimate peaked at 1.6 at 17 days post-abscess and gradually declined to 1.0 by day 200, reaching a nadir of 0.92 around day 300, before increasing again thereafter (Fig. 1). However, for follow-up times beyond approximately 100 days, all confidence intervals included 1.0, indicating that the apparent late increase was not statistically significant. A similar but less pronounced trend was observed in the subgroup with non-skin abscesses, though the ratio never differed statistically from 1.

**Table 2:** Plasma anti-αGal antibody concentration in healthy individuals before and after an abscess event

			Mean anti-αGal in mg/L (95% CI)			
**Stratification**		**Cases**, ***n***	**Before abscess**	**After abscess**	**Concentration ratio (95% CI)**	**P**
All:		106	11 (8.4, 15)	12 (9.3, 16)	1.1 (0.97, 1.2)	0.14
Sex:						
	Females	46	13 (8.4, 20)	15 (10, 21)	1.2 (0.97, 1.4)	0.08
	Males	60	10 (7.1, 15)	10 (7.1, 16)	1.0 (0.89, 1.2)	0.78
Age:						
	17–43 yrs.	53	12 (7.8, 17)	14 (9.4, 19)	1.2 (0.99, 1.3)	0.07
	44–67 yrs.	53	11 (7.2, 16)	11 (7.4, 16)	1.0 (0.87, 1.1)	0.94
ABO blood group:						
	B or AB	16	3.9 (1.8, 9.9)	4.9 (2.6, 9.7)	1.3 (0.80, 1.8)	0.35
	O or A	90	14 (10, 18)	14 (11, 19)	1.1 (0.95, 1.2)	0.31
Abscess site						
	Skin	63	9.5 (6.4, 14)	11 (7.6, 16)	1.2 (0.98, 1.3)	0.08
	Non-skin	43	14 (10, 20)	14 (9.7, 21)	0.99 (0.88, 1.1)	0.84
Time from abscess to follow-up sample collection:						
	< 187 days	53	12 (7.8, 18)	14 (9.6, 20)	1.2 (1.0, 1.4)	0.03
	≥ 187 days	53	11 (7.4, 16)	10 (7.2, 15)	0.97 (0.85, 1.1)	0.66

Non-skin abscesses were the oral cavity (*n* < 5), deep neck (*n* < 5), or anal region (*n* = 38). The 95% CIs were calculated by bootstrap with 5000 iterations. The p-values represent the likelihood that the average concentration ratio between after and before the abscess event differs from 1 and were calculated using a two-sided permutation test with 5000 iterations

As a control, we compared anti-αGal antibody concentrations in baseline samples from cases to those from individually matched controls without abscess diagnoses. No significant differences were observed in the paired analyses (**Supplementary Table 3**).

These results suggest that a single skin abscess event may be followed by a temporary increase in plasma anti-αGal antibody levels.

### High burden of skin abscesses is associated with elevated levels of anti-αGal antibody

To further assess the impact of skin abscesses on anti-αGal antibody levels, we conducted an additional substudy in the Recurrent Skin Abscess Cohort. This cohort included 43 patients with a history of recurrent skin abscesses, 75 patients with a history of other types of infections (serving as controls), and 60 healthy blood donors as a further control group.

We found that the average concentration of plasma anti-αGal antibodies in patients with recurrent skin abscesses was 16 mg/L (95% CI: 11–22 mg/L) compared with 8.7 mg/L (95% CI: 6.0–13 mg/L) in patient controls and 7.5 mg/L (95%CI: 5.4–10 mg/L) in healthy controls (Fig. 2). Thus, the mean anti-αGal antibody level in patients with skin abscesses was 1.8-fold (95% CI: 1.1–2.9) higher than in the patient controls and 2.1-fold (95% CI: 1.4–3.3) higher than in the healthy controls. Visual examination of the data suggests that the groups primarily differed in the proportion of participants with lower concentrations of the antibodies. Notably, no patients with recurrent skin abscesses had antibody concentrations below 2.5 mg/L, whereas this was true for 17% of the patient controls and 15% of the healthy controls.

Analyses stratified by sex, age relative to the median of all individuals in this cohort (median: 37 years), and B-antigen expression, revealed that the relative antibody concentration between patients with skin abscesses and the two control groups had point estimates of 1.6 or higher in 11 out of 12 defined subgroups (Fig. 3). The only exception was observed in the comparison between patients with recurrent skin abscesses and patient controls older than 37 years, where the relative antibody concentration was 1.0 (95% CI: 0.53–2.2). This difference appeared to be driven by higher average antibody concentrations in patient controls compared to the other stratified analyses. The elevated average was attributable to a subset of older patient controls with particularly high concentrations. In a sensitivity analysis using linear regression, a positive association between age and anti-αGal antibody levels was observed in patient controls (Supplementary Fig. S1), which might suggest that longer exposure to various infections with increasing age may lead to higher plasma levels of anti-αGal antibodies in this group.

These results suggest that individuals with repeated skin abscesses may have significantly increased levels of plasma anti-αGal antibodies.

## Discussion

In this study, we investigated the impact of bacterial colonization and common infections on naturally occurring antibody levels in humans, with a specific focus on anti-αGal antibodies, using *S. aureus* as the model organism. Our findings suggest that nasal colonization by *S. aureus* is negatively associated with plasma concentrations of anti-αGal antibodies, whereas skin abscesses, primarily caused by *S. aureus*, are followed by increased levels of these antibodies.

TRIFMA was chosen over ELISA for antibody measurements due to its higher sensitivity, broader dynamic range, and improved signal stability [17, 36–38]. By utilizing lanthanide chelates as fluorescent labels, TRIFMA reduces background noise and enhances detection limits, making it particularly suitable for measuring low-abundance antibodies and detecting subtle changes over time. Additionally, TRIFMA offers a more stable readout with lower variability, ensuring greater reproducibility in our analyses.

The previously reported reactivity of anti-αGal antibodies with *S. aureus* [20] does not necessarily indicate that this organism expresses or displays αGal antigens. The αGal epitope is exceptionally rare in bacteria [39–41] and has not been detected in selected *S. aureus* strains [41]. A more plausible explanation for this reactivity is the synergistic polyreactivity exhibited by anti-αGal antibodies [1]. These antibodies consist of multiple clones with distinct specificities beyond their shared αGal reactivity. Through synergistic interactions, these clones achieve broad-spectrum antigen reactivity [1]. In pilot experiments, we have observed minimal binding to well-characterized mutants of the Reynolds strain [42] expressing capsule serotypes 5 or 8, suggesting that these capsular polysaccharides are not significant antigens for these antibodies (data not shown).

Our a priori hypothesis was that nasal *S. aureus* colonization would be associated with higher anti-αGal antibody levels due to increased exposure to the bacterium. However, contrary to this expectation, we found that nasal *S. aureus* colonization was associated with 35% lower plasma levels of anti-αGal antibodies compared to non-colonized individuals. Although this unexpected result could represent a type I statistical error, it appears robust given its consistency across stratified analyses. One potential explanation is that *S. aureus* colonization may actively reduce plasma anti-αGal antibody levels, either by inducing partial immune tolerance or by displacing other microorganisms that more strongly stimulate these antibodies. Since anti-αGal antibodies are thought to contribute to antimicrobial defense [1] and correlate with broader pathogen-reactive antibody levels [11, 12], another possibility is that higher levels of these antibodies confer protection against *S. aureus* colonization, or reflect underlying mucosal immunity. However, the cross-sectional design of this substudy precludes causal inference, and these interpretations should be considered speculative.

We observed that a single skin abscess event led to a 30% increase in plasma anti-αGal antibody levels within 187 days post-infection. The 187-day cutoff was chosen for statistical reasons, as it represents the median follow-up time, ensuring balanced group sizes and minimizing the impact of extreme values. Although not selected based on specific clinical considerations, it provides a standardized approach for assessing antibody responses post-abscess. Future research may explore optimal follow-up timing in relation to clinical outcomes. The 30% increase in plasma anti-αGal antibody levels may seem modest, but it is noteworthy given that only a small subset (in the order of 1%) of anti-αGal antibody clones is expected to exhibit polyreactivity toward a reactive but non-αGal-presenting pathogen targeted by these antibodies [1]. For such a small subset to account for the observed increase in total anti-αGal antibody levels, its concentration must increase disproportionately. Specifically, if this subset originally constituted 1% of the total anti-αGal antibodies, then a 30-fold expansion of this subset would be required to drive a 30% increase in the overall antibody pool. This calculation, detailed in **Supplementary Table 4**, highlights the potential magnitude of expansion required for a polyreactive subset to significantly influence total anti-αGal levels.

No statistically significant increase in antibody levels was observed in samples collected after 187 days, suggesting that the rise in anti-αGal antibodies is transient following a single exposure and subsequently declines. Additionally, non-skin abscesses, such as anal, oral, and deep neck abscesses, did not correlate with increased anti-αGal antibody levels. *S. aureus* is more rarely involved in such abscesses [26, 27, 43–45]. The pathogens causing these non-skin abscesses may lack reactivity with anti-αGal antibodies, which could explain why these infections did not result in elevated antibody levels. A limitation of this substudy is the lack of access to the actual pathogenic organisms, which prevented us from directly examining their nature or their reactivity with anti-αGal antibodies. Thus, we cannot rule out the possibility that pathogens other than *S. aureus* may have contributed to the induction of anti-αGal antibodies.

Our study also demonstrated that patients with recurrent skin abscesses had higher plasma levels of anti-αGal antibodies compared with controls, further suggesting that skin abscesses significantly influence anti-αGal antibody levels. Limitations in this substudy include its cross-sectional design, lack of case-control matching, and the small sample size for the stratified analysis of B-antigen-expressing individuals, with only two cases available for analysis. Conversely, our substudy of single abscess events in healthy individuals was more robust, utilizing paired samples taken before and after the abscess event. This design is unique and provides valuable insight. A limitation to that substudy is the reliance on registry-based abscess diagnoses without validation, though the registry used is considered highly reliable [33].

While this study focuses on bacterial exposures as drivers of naturally occurring antibodies, specifically IgG anti-αGal antibodies, we acknowledge that specific host-related factors may be of particular importance for the production of some naturally occurring antibodies, but not necessarily others. In the context of skin abscesses, *S. aureus*, and anti-αGal antibodies, atopy may be particularly relevant. Atopic individuals are at increased risk of *S. aureus* skin infections [46] and may also be more prone to producing IgE anti-αGal antibodies [47], which could be important in future investigations.

In conclusion, this study suggests that common infections help shape the repertoire of naturally occurring antibodies in humans. The observation of lower anti-αGal antibody levels in *S. aureus* nasal carriers might imply an association between reduced systemic anti-αGal levels and colonization. Further studies are warranted to clarify this relationship and to better understand the role of anti-αGal antibodies in mucosal immunity.

## Electronic supplementary material

Below is the link to the electronic supplementary material.

Supplementary Material 1

## Data Availability

Data relating to the manuscript will be available upon reasonable request to the corresponding author.
