# Supplementary material for: Impact of Staphylococcus aureus colonization and skin abscesses on formation of human anti-αGal antibodies
Source: Med Microbiol Immunol. 2025 May 13;214(1):23. doi: 10.1007/s00430-025-00833-3 (PMC12075334; doi:10.1007/s00430-025-00833-3)
Supplement: Supplementary file 1 — Supplementary Material 1 [file 430_2025_833_MOESM1_ESM.pdf]

Impact of *Staphylococcus aureus* colonization and skin abscesses on formation of human anti-αGal antibodies

Supplementary Material

Supplementary Table 1. The following abscess diagnoses based on the International Classification of Diseases, 10th edition (ICD-10) were included when occurring as primary diagnosis, secondary diagnosis, and/or underlying medical condition.

NPR code	Text (Danish)	ICD-10 code	Text (English)	Cases, *n*
H000A	Absces øjenlåg	H00.0	Hordeolum and other deep inflammation of eyelid (abscess)	0
H050A	Absces øjenhule	H05.0	Acute inflammation of orbit (abscess)	0
H440A	Absces glaslegeme	H44.0	Purulent endophthalmitis (Vitreous abscess)	0
H6000+A	Absces i ydre øre og øregang	H60.0	Abscess of external ear (Boil)	0
J340A	Absces i næsen	J34.0	Abscess, furuncle and carbuncle of nose	0
J36	Absces i hals	J36	Peritonsillar abscess	0
J390	Retro- eller parafaryngeal abscess	J39.0	Retropharyngeal and parapharyngeal abscess	< 5
J391	Absces pharynx (inkl. undergrupper)	J39.1	Other abscess of pharynx	0
K130A	Absces i labii oris	K13.0	Diseases of lips (Cheilitis)	0
K122A+B	Absces submandibularis og abscess i munden	K12.2	Cellulitis and abscess of mouth (Cellulitis of mouth (floor) OR Submandibular abscess)	< 5
K113	Absces i spytkirtler	K11.3	Abscess of salivary gland	0
K610A	Perianalabsces	K61.0	Anal abscess	38
K614	Intrasfinkterisk analabsces	K61.4	Intrasphincteric abscess	0
L02C	Hudabsces UNS	L02	Cutaneous abscess, furuncle and carbuncle (unspecified)	0
L020C	Absces i ansigtet	L02.0	Cutaneous abscess, furuncle and carbuncle of face	0
L021C	Absces på hals	L02.1	Cutaneous abscess, furuncle and carbuncle of neck	0
L022N	Absces på ryggen	L02.2	Cutaneous abscess, furuncle and carbuncle of trunk (back [any part, except buttock])	7
L022P	Absces på kroppen UNS	L02.2	Cutaneous abscess, furuncle and carbuncle of trunk (unspecified)	< 5
L022 (O+Q+R)	Absces i navlen, perineum eller bugvæggen.	L02.2	Cutaneous abscess, furuncle and carbuncle of trunk (Umbilicus, Perineum, or Abdominal wall)	0
L022S	Absces på brystvæggen	L02.2	Cutaneous abscess, furuncle and carbuncle of trunk (Chest wall)	< 5
L022T	Absces i lysken	L02.2	Cutaneous abscess, furuncle and carbuncle of trunk (Groin)	8
L023C	Absces i sæderegion	L02.3	Cutaneous abscess, furuncle and carbuncle of buttock	< 5
L024	Abscesser i huden på ekstremiteter (alle undergrupper)	L02.4	Cutaneous abscess, furuncle and carbuncle of limb	37
L028C	Abscesser på hoved	L02.8	Cutaneous abscess, furuncle and carbuncle of other sites (head)	< 5
L88 (ekskl. L889 & L889C)	Pusdannelse i huden ekskl. 'Pyoderma gangraenosum' & 'pyodermia phagedenica.'	Not defined	Accumulation of purulent material in skin excluding 'Pyoderma gangrenosum' and 'Phagedenic pyoderma'	0
**Total**				**106**

In red: NPR codes for abscess types with case numbers > 0 where *Staphylococcus aureus* is an unusual cause (J390 [1], K122A+B [2, 3], and K610A [4, 5]). These diagnoses were categorized as non-skin abscesses.

The use of '<5' for small numbers adheres to anonymization requirements to protect participant privacy.

Supplementary Data File: Impact of *Staphylococcus aureus* colonization and skin abscesses on formation of human anti-αGal antibodies

Supplementary Table 2. Plasma anti-αGal antibody concentration in healthy individuals before and after abscess according to follow-up time.

		Mean anti-αGal in mg/L (CI)			
**Stratification**	**Cases, *n***	**Before abscess**	**After abscess**	**Relative concentration (CI)**	**P**
Females					
< 187 days	24	13 (7.3, 24)	16 (11, 25)	1.3 (0.94, 1.6)	0.09
≥ 187 days	22	12 (6.9, 23)	13 (7.8, 24)	1.1 (0.86, 1.3)	0.51
Males					
< 187 days	29	11 (6.1, 20)	13 (7.2, 22)	1.2 (0.95, 1.4)	0.13
≥ 187 days	31	9.8 (6.0, 16)	8.8 (5.4, 15)	0.9 (0.77, 1.1)	0.18
Age 17-43 years					
< 187 days	28	12 (7.0, 22)	15 (9.2, 25)	1.2 (0.95, 1.5)	0.09
≥ 187 days	25	11 (6.5, 19)	12 (7.3, 20)	1.1 (0.89, 1.3)	0.42
Age 44-67 years					
< 187 days	25	11 (6.0, 20)	13 (7.5, 22)	1.2 (0.94, 1.4)	0.15
≥ 187 days	28	11 (6.2, 19)	9.2 (5.4, 16)	0.87 (0.75, 1.0)	0.09
ABO blood group B or AB:					
< 187 days	6	2.1 (0.86, 7.3)	4.5 (3.3, 6.7)	2.1 (0.84, 4.3)	0.12
≥ 187 days	10	5.6 (2.0, 19)	5.2 (2.0, 15)	0.92 (0.7, 1.2)	0.54
ABO blood group O or A:					
< 187 days	47	15 (9.7, 22)	16 (11, 24)	1.1 (0.98, 1.3)	0.09
≥ 187 days	43	13 (8.6, 18)	12 (8.3, 18)	0.98 (0.85, 1.1)	0.81
Non-skin abscess					
< 187 days	22	14 (7.9, 24)	14 (8.4, 26)	1.0 (0.87, 1.3)	0.66
≥ 187 days	21	15 (9.5, 24)	14 (8.4, 24)	0.93 (0.81, 1.1)	0.34
Skin abscess					
< 187 days	31	10 (5.8, 19)	14 (8.5, 22)	1.3 (1.0, 1.7)	0.01
≥ 187 days	32	8.7 (5.1, 15)	8.6 (5.3, 15)	0.99 (0.82, 1.2)	0.96

The 95% confidence intervals (CI) were calculated by bootstrap with 5000 iterations. The p-values are the likelihood that the average relative concentration differs from 1 and were calculated by a two-sided permutation test with 5000 iterations.

Supplementary Table 3. Plasma anti-αGal antibody concentration in healthy individuals with later abscess (cases) and individuals without abscess (controls).

		Mean anti-αGal in mg/L (CI)			
**Stratification**	**Pairs, *n***	**Case (before abscess)**	**Control**	**Relative concentration (CI)**	**P**
All:	106	11 (8.5, 15)	11 (8.9, 14)	0.99 (0.67, 1.5)	0.96
Sex:					
Females	46	13 (8.4, 19)	10 (7.5, 14)	1.22 (0.71, 2.1)	0.47
Males	60	10 (7.0, 15)	12 (8.5, 18)	0.84 (0.48, 1.5)	0.56
Age:					
17–43 yrs.	53	12 (7.9, 17)	9.5 (6.9, 13)	1.22 (0.75, 2.0)	0.43
44–67 yrs.	53	11 (7.1, 16)	13 (9.4, 19)	0.81 (0.43, 1.5)	0.49
ABO blood group:					
B or AB	16	3.9 (1.7, 9.2)	7.4 (3.9, 13)	0.52 (0.16, 1.8)	0.32
O or A	90	14 (10, 18)	12 (9.4, 16)	1.11 (0.74, 1.7)	0.62

The 95% confidence intervals (CI) were calculated by bootstrap with 5000 iterations. The p-values are the likelihood that the average relative concentration differs from 1 and were calculated by a two-sided permutation test with 5000 iterations.
The limits of the CI 'Before abscess' are not exactly the same as in Table 2 (main text) as the bootstrap calculations were made separately for the two tables.

Supplementary Data File: Impact of *Staphylococcus aureus* colonization and skin abscesses on formation of human anti-αGal antibodies

Supplementary Fig. 1. Association between plasma anti-αGal antibody concentration and age in recurrent skin abscess patients and patient controls

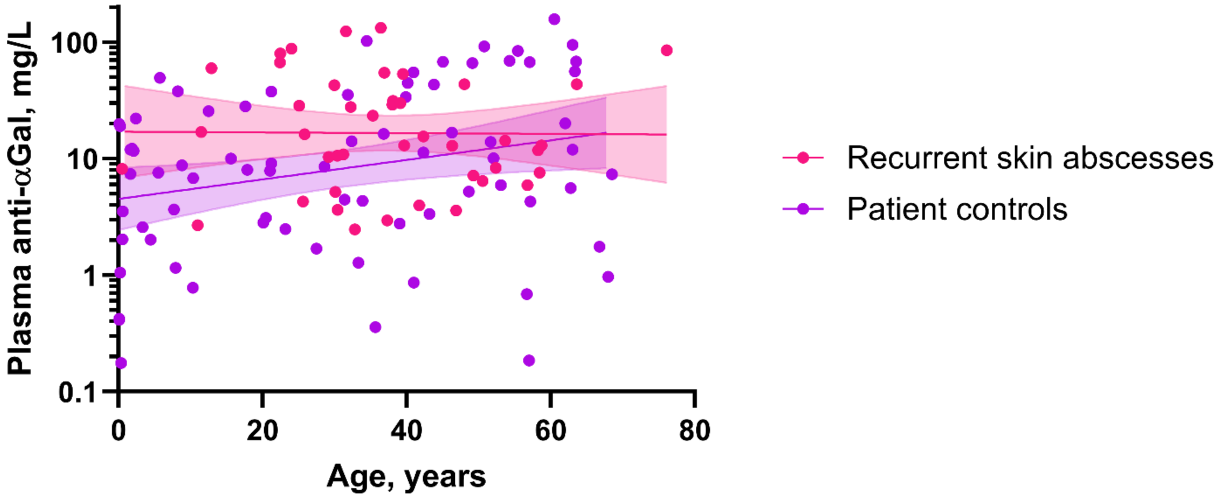

Data from Fig. 3 in the main text are presented, showing antibody levels as a function of age. Linear regression analysis of log10-transformed antibody levels revealed no significant difference in slopes between the two groups: -0.00031 (95% CI: -0.010–0.0097) for patients with recurrent skin abscesses and 0.0084 (95% CI: 0.0013–0.015) for patient controls (P = 0.22). However, the slope for the patient control group was significantly greater than zero, indicating a positive association between age and antibody levels. This suggests that longer exposure to various infections, as individuals age, may increase plasma levels of anti-αGal antibodies in the control group.

Supplementary Table 4. The required increase in relative concentration of an antibody subset comprising 1% of the total antibodies to achieve a 30% increase in total antibody concentration.

If total concentration of anti-αGal antibodies 'C_t' increase by 30% after abscess relative to their concentration before, then:

$$C_{t,after} = 1.3 \times C_{t,before}$$

If total anti-αGal antibodies contain one subset denoted 'αS' and the remaining antibodies are denoted '$other$', then:

$$C_{\alpha S,after} + C_{other,after} = 1.3 \times \left(C_{\alpha S,before} + C_{other,before}\right)$$

If only the concentration of the αS antibody subset is influenced by the abscess, then:

$$C_{\alpha S,after} + C_{other,before} = 1.3 \times \left(C_{\alpha S,before} + C_{other,before}\right)$$

If the αS antibody subset comprised 1% of the total anti-αGal antibodies before abscess (i.e., $C_{other,before} = 99 \times C_{\alpha S,before}$), then:

$$C_{\alpha S,after} + 99C_{\alpha S,before} = 1.3 \times \left(C_{\alpha S,before} + 99C_{\alpha S,before}\right) = 1.3 \times 100C_{\alpha S,before}$$

Thus:

$$C_{\alpha S,after} = 130C_{\alpha S,before} - 99C_{\alpha S,before} = 31C_{\alpha S,before}$$

And:

$$\frac{C_{\alpha S,after}}{C_{\alpha S,before}} = 31$$

Thus, the αS subset is required to increase 31-fold in concentration to cause a 30% increase in total anti-αGal antibody concentration.

References:

1. Ridder, G.J., et al., *Spectrum and management of deep neck space infections: an 8-year experience of 234 cases.* Otolaryngol Head Neck Surg, 2005. **133**(5): p. 709-14. https://doi.org/10.1016/j.otohns.2005.07.001.
2. Boscolo-Rizzo, P. and M.C. Da Mosto, *Submandibular space infection: a potentially lethal infection.* Int J Infect Dis, 2009. **13**(3): p. 327-33. https://doi.org/10.1016/j.ijid.2008.07.007.
3. Rega, A.J., S.R. Aziz, and V.B. Ziccardi, *Microbiology and antibiotic sensitivities of head and neck space infections of odontogenic origin.* J Oral Maxillofac Surg, 2006. **64**(9): p. 1377-80. https://doi.org/10.1016/j.joms.2006.05.023.
4. Bender, F., et al., *Drug resistant bacteria in perianal abscesses are frequent and relevant.* Sci Rep, 2022. **12**(1): p. 14866. https://doi.org/10.1038/s41598-022-19123-6.
5. Liu, C.K., et al., *Clinical and microbiological analysis of adult perianal abscess.* J Microbiol Immunol Infect, 2011. **44**(3): p. 204-8. https://doi.org/10.1016/j.jmii.2011.01.024.
